# Dynamic and reversible DNA methylation changes induced by genome separation and merger of polyploid wheat

**DOI:** 10.1186/s12915-020-00909-x

**Published:** 2020-11-20

**Authors:** Jingya Yuan, Wu Jiao, Yanfeng Liu, Wenxue Ye, Xiue Wang, Bao Liu, Qingxin Song, Z. Jeffrey Chen

**Affiliations:** 1 State Key Laboratory of Crop Genetics and Germplasm Enhancement, Nanjing Agricultural University, 1 Weigang Road, Nanjing, 210095 China; 2 Key Laboratory of Molecular Epigenetics of the Ministry of Education, Northeast Normal University, Changchu, 130024 China; 3 Department of Molecular Biosciences, The University of Texas at Austin, Austin, TX 78712 USA

**Keywords:** Extracted tetraploid wheat, DNA methylation, Transposon, Genomics, Polyploidy, Wheat evolution

## Abstract

**Background:**

Wheat is a powerful genetic model for studying polyploid evolution and crop domestication. Hexaploid bread wheat was formed by two rounds of interspecific hybridization and polyploidization, processes which are often accompanied by genetic and epigenetic changes, including DNA methylation. However, the extent and effect of such changes during wheat evolution, particularly from tetraploid-to-hexaploid wheat, are currently elusive.

**Results:**

Here we report genome-wide DNA methylation landscapes in extracted tetraploid wheat (ETW, AABB), natural hexaploid wheat (NHW, AABBDD), resynthesized hexaploid wheat (RHW, AABBDD), natural tetraploid wheat (NTW, AABB), and diploid (DD). In the endosperm, levels of DNA methylation, especially in CHG (H=A, T, or C) context, were dramatically decreased in the ETW relative to natural hexaploid wheat; hypo-differentially methylated regions (DMRs) (850,832) were 24-fold more than hyper-DMRs (35,111). Interestingly, those demethylated regions in ETW were remethylated in the resynthesized hexaploid wheat after the addition of the D genome. In ETW, hypo-DMRs correlated with gene expression, and TEs were demethylated and activated, which could be silenced in the hexaploid wheat. In NHW, groups of TEs were dispersed in genic regions of three subgenomes, which may regulate the expression of TE-associated genes. Further, hypo-DMRs in ETW were associated with reduced H3K9me2 levels and increased expression of histone variant genes, suggesting concerted epigenetic changes after separation from the hexaploid.

**Conclusion:**

Genome merger and separation provoke dynamic and reversible changes in chromatin and DNA methylation. These changes correlate with altered gene expression and TE activity, which may provide insights into polyploid genome and wheat evolution.

## Background

Polyploidy is a prominent feature for the evolution of some animals and all flowering plants, including most important crops such as wheat, cotton, and canola [1–3]. The common occurrence of polyploidy suggests an advantage for having additional genetic materials for diversification of polyploid plants and domestication of polyploid crops. Interspecific hybridization in cotton is accompanied with genetic and epigenetic changes including DNA methylation, many of which are maintained as stable epialleles among five allotetraploid cotton species during evolution and two cultivated cottons during domestication [4]. In addition to DNA methylation, genome-wide histone modifications determine expression bias of homeologous genes in alloptetraploid cotton [5], and long-noncoding RNAs (lncRNAs) are predominantly transcribed from transposable element (TE) regions that are demethylated in a cotton interspecific hybrid [6]. This is consistent with the nonadditive expression of microRNAs (miRNAs) and small interfering RNAs (siRNAs) in *Arabidopsis* allotetraploids as a response to “genome shock” during interspecific hybridization [7].

**Fig. 1:**
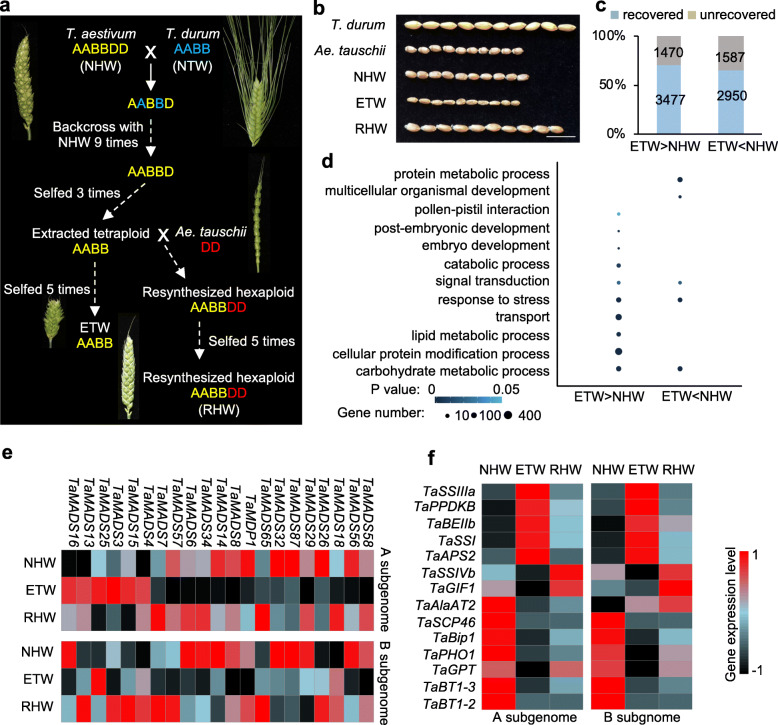
Differentially expressed genes between extracted tetraploid wheat (ETW, AABB) and natural hexaploid wheat (NHW, AABBDD). **a** Diagram for producing ETW from NHW, modified from those of [13, 14]. The hybrid (AABBD) between NHW and NTW was backcrossed with NHW for nine generations, followed by selfing for three generations to select AABB genotype, which was selfed for additonal five generations to maintain genome stability. The resynthesized hexaploid was made between ETW (AABB) and DD and self-pollinated for five generaitons. **b** Mature seed morphology of *T. durum*, *Ae. tauschii*, NHW, ETW, and resynthesized hexaploid wheat (RHW, AABBDD). Scale bar = 1 cm. **c** Number of differentially expressed genes (DEGs) between ETW and NHW, with the majority (blue) restored and fewer not restored (yellow) in RHW. **d** Gene ontology (GO) analysis of DEGs. The circle color and size indicate *P* value and gene number, respectively (hypergeometric test, *P* < 0.05). **e**, **f** Heatmaps showing expression levels (fragment per kiobase per million, FPKM) of MADS-box genes (**e**) and starch biosynthetic genes (**f**) in ETW, NHW, and RHW

The evolution of hexaploid bread wheat (*Triticum aestivum* L.) coincided with human civilization through two rounds of interspecific hybridization and whole-genome duplication (WGD) [8, 9], involving mechanisms of correct pairing between homoeologous chromosomes [10]. The common wheat (2n=6x=42, AABBDD) was formed ~ 8000–10,000 years ago by crossing between tetraploid wheat (*Triticum turgidum* L., AABB) and a goatgrass species (*Aegilops tauschii*, DD), coupled with genome doubling [8]. The A, B, and D subgenomes remain largely intact, with only a few homoeologous chromosome translocations [11, 12]. The tetraploid genomic (AABB) component could be extracted by hybridization between a natural hexaploid wheat (NHW, AABBDD) and a natural tetraploid wheat (NTW, AABB) with nine generations of backcrossing using NHW as the recurrent parent, followed by three generations of self-pollination to exclude D chromosome and stabilize the genotype [13] (Fig. 1a). The extracted tetraploid wheat (ETW, AABB) is dwarfed and displays severe developmental abnormalities compared to natural tetraploid and hexaploid wheat, but the chromosomes pair and segregate normally [13, 14]. The abnormal phenotypes are recovered in the resynthesized allohexaploid wheat (RHW) formed by hybridization between ETW and a D-genome diploid (*Ae. tauschii*) [13, 14]. Previous studies show that ETW has lower levels of DNA methylation using 5-methylC antibodies [15] and more differentially expressed genes [14], compared to the natural tetraploid wheat (NTW). However, it is unknown how DNA methylation changes from the separation of the ETW (AABB component) in the natural hexaploid wheat to the formation of resynthesized hexaploid wheat.

In this study, we performed RNA sequencing (RNA-seq) and whole-genome bisulfite sequencing (BS-seq) in the endosperm of ETW (AABB), the natural hexaploid wheat (*T. aestivum* L. cv. Canthach), natural tetraploid wheat (*T. turgidum* L. subsp. *durum*), *Ae. tauschii* subsp. *strangulata* (line RL5288, DD), and the resynthesized hexaploid wheat. We found genome-wide reduction of DNA methylation in the ETW and remethylation of these demethylated regions in the resynthesized hexaploid wheat after addition of the D subgenome, which are accompanied by changes in gene and TE expression. DNA demethylation in ETW coincides with reduced H3K9me2 levels and increased expression of histone genes. These results suggest that genome separation and merger promote dynamic and reversable epigenetic and gene expression changes in the evolution of polyploid wheat.

## Results

### Phenotypic, karyotypic, and gene expression changes in ETW

ETW was produced by hybridization between a natural hexaploid wheat (NHW, AABBDD) and a natural tetraploid wheat (NTW, AABB). The resulting pentaploid (AABBD) was backcrossed to NHW for nine generations, followed by three generations of self-pollination to stabilize the AABB karyotype and exclude D chromosomes (Fig. 1a) [13]. After separation of ETW (AABB) from the DD in NHW (AABBDD), ETW displays severe phenotypic defects, including dwarfed plants, compacted spikes, shriveled seeds, decreased starch content, smaller starch granule, and reduced fertility [14] (Fig. 1a and Additional file 1: Figure S1). Interestingly, the seed size and other abnormal phenotypes were recovered in the resynthesized allohexaploid wheat (RHW) by addition of the D subgenome (*Ae. tauschii*) to ETW (Fig. 1b). Fluorescent in situ hybridization (FISH) analysis showed stable karyotypes of homoeologous chromosomes in ETW relative to NHW and NTW (Additional file 1: Figure S2), which is consistent with the notion that ETW is highly (> 99.8%) identical to the AB subgenomes of its NHW donor after the seventh [13] or ninth backcross [14] (Fig. 1a).

The phenotypic variation such as seed size in ETW could be related to gene expression changes in developing seeds. In wheat, the development of starchy endosperm and aleurone layer begins from ~ 6 days after pollination (DAP) and is critical to seed development [16]. At this stage, seed size and shape were noticeably smaller in ETW than in NHW and RHW (Additional file 1: Figure S1b). To determine gene expression changes during seed development, we performed RNA-seq analysis in the endosperm (6 DAP) of ETW, NHW, RHW, NTW (*T. durum*), and DD diploid (*Ae. tauschii*) (Additional file 2: Table S1). Between ETW and NHW, 4947 and 4537 genes were up- and downregulated, respectively (Additional file 2: Table S2). Of these differentially expressed genes (DEGs), the majority (65~70%) were recovered in the RHW after the addition of the D genome to ETW (Fig. 1c). Gene ontology (GO) analysis showed overrepresentation of the DEGs in metabolism and development-related processes, including protein metabolism and embryo development (Fig. 1d), suggesting a potential role of these pathway genes in seed phenotypic changes in ETW after separation from NHW. For example, in *Arabidopsis* and rice, MADS-box genes are expressed in developing seeds and play important roles in reproductive development [17, 18]. Knockdown of *OsMADS7* and *OsMADS8* could induce variable floral organs in rice [19]. These MADS-box genes such as *TaMADS8* and *TaMADS7* were repressed in ETW and derepressed in RHW after adding back the D subgenome (Fig. 1e), indicating a possible role of MADS-box genes in seed size reduction in ETW. Consistent with low starch content and small kernel in the ETW (Additional file 1: Figure S1c, d), the genes involved in starch biosynthesis were also downregulated (Fig. 1f).

### Genome-wide demethylation in ETW and remethylation after regain of DD in RHW

**Fig. 2:**
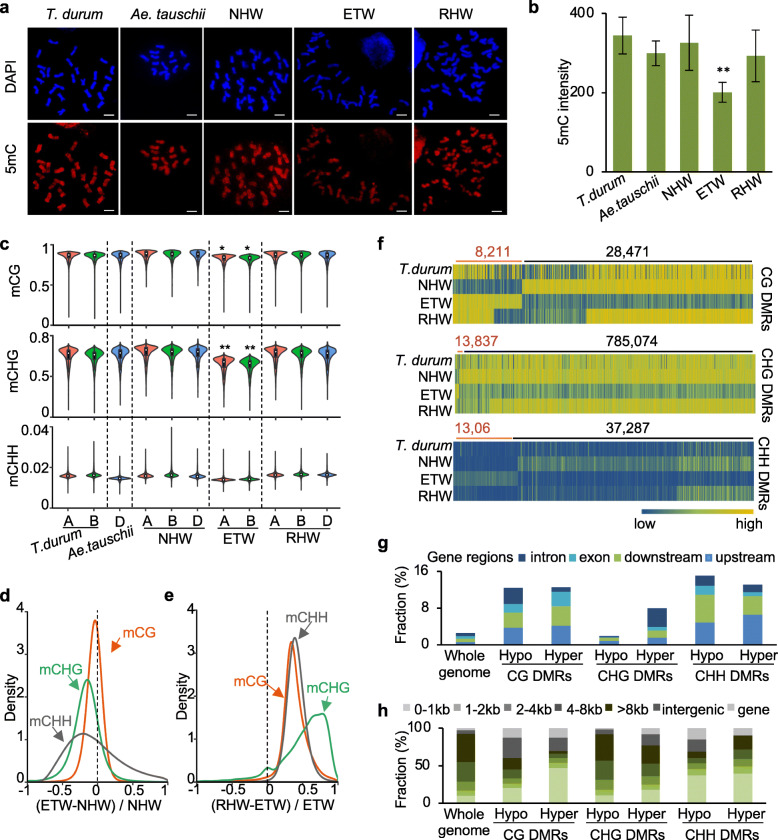
DNA methylation landscapes in different wheat species. **a**, **b** Representative micrographs (**a**) and relative intensities (**b**) of 5mC immunofluorescence images in metaphase cells of different wheat species. Error bars indicate standard deviation in 30 cells (*n* = 30) with double asterisks showing a significance level of *P* < 0.01. Scale bar = 10 μm. **c** Violin plots showing genome-wide DNA methylation distributions of A (red), B (green), and D (blue) subgenomes in *T. durum*, *Ae. tauschii*, NHW, ETW, and RHW (Wilcoxon rank-sum test, single and double asterisks indicate significance levels of *P* < 0.05 and *P* < 0.01, respectively). **d**, **e** Kernel density plots displaying CG, CHG, and CHH methylation changes between ETW and NHW (**d**) and between RHW and ETW (**e**) using a 200-bp window. **f** Differentially methylated regions (DMRs) in CG (upper), CHG (middle), and CHH (lower panel) between ETW and NHW in NTW (*durum*), NHW, ETW, and RHW. The scales are 0–1 for CG and CHG level and 0–0.05 for CHH. **g** Percentage of DMRs in genic regions: intron, exon, upstream, and downstream. **h** Percentage of DMRs in TE regions: 0–1, 1–2, 2–4, 4–8 kb, and > 8 kb

Similar to phenotypic and gene expression changes, overall DNA methylation levels of immunofluorescence-stained chromosomes were decreased in the ETW and returned to the same status after gaining the D subgenome in the resynthesized allohexaploid wheat (RHW) as in NHW (Fig. 2a, b). To reveal DNA methylation changes before and after genome separation and merger, we performed whole-genome methylcytosine sequencing (MethylC-seq) in the endosperm of ETW, NHW, RHW, NTW (*T. durum*), and diploid (*Ae. tauschii*, DD) lines (Additional file 2: Table S3). To avoid potential effects of genomic variation on MethylC-seq analysis, we resequenced *T. durum*, *Ae. tauschii*, ETW, and NHW (Additional file 2: Table S4) and identified 28,536,651, 9,059,622, 18,513,346, and 23,414,348 SNPs, as well as 1,721,347, 895,291, 1,165,534, and 1,595,081 insertions/deletions (Indels), respectively, relative to the Chinese Spring genome (WGAv1) [11]. In addition, a total of 25,774 copy number variations (CNVs) were identified in all lines relative to the Chinese Spring genome (WGAv1). MethylC-seq reads were mapped to the SNP-corrected Chinese Spring genome (WGAv1), excluding those in the Indel and CNV regions, for further analysis. The bisulfite-conversion rate was > 99% (Additional file 2: Table S3), with a mean coverage of cytosines at ~ 8.8-fold of genome equivalent for all genotypes (Additional file 2: Table S3). Consistent with decreased methylation levels by immunofluorescence analysis, CG and CHG methylation levels were decreased in both A and B subgenomes of ETW compared to NHW (Fig. 2c–e and Additional file 1: Figure S3a, 3b) (Wilcoxon rank-sum test, *P* < 0.05), while the methylation levels were recovered in the RHW, to a similar level as in the NHW (Fig. 2c–e).

Further analysis identified predominately hypo-differentially methylated regions (hypo-DMRs) between ETW and NHW in CG (28,471), CHG (785,074), and CHH (37,287) sites (Fig. 2f), whereas hyper-DMRs were substantially lower in all contexts, including 8211 (CG), 13,837 (CHG), and 13,063 (CHH), respectively (Fig. 2f). Among hypo-DMRs, the majority in CG (22,340, 78.4%) and CHG (707,665, 90.1%) plus one-third in CHH (12,308, 33.0%) were reverted to higher levels in the RHW (Fig. 2f and Additional file 2: Table S5). All CG and CHH DMRs, except for CHG hypo-DMRs, were enriched in genic regions (Fig. 2g), suggesting their potential roles in gene expression. For TE regions, CG hyper-DMRs and CHH DMRs were enriched in short TEs (Fig. 2h). The distribution pattern of CHG hypo-DMRs was similar to the whole-genome methylation profile in both genic and TE regions (Fig. 2g, h), indicating global demethylation of CHG context throughout two subgenomes in the ETW (Additional file 1: Figure S3b).

**Fig. 3:**
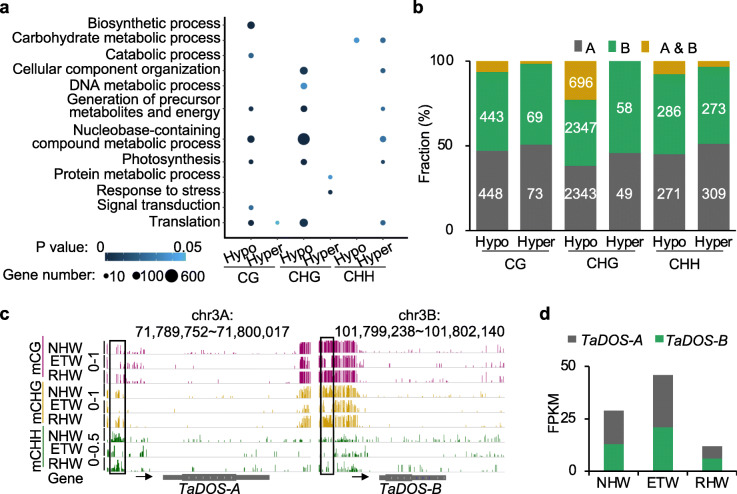
Relationship between gene expression and DNA methylation in ETW. **a** GO representation of DMR-associated genes in ETW. The circle color and size indicate *P* value and gene number, respectively. **b** DNA methylation changes in A (gray), B (green), and both A and B (yellow) homoeologous genes in ETW. **c** An example showing DNA methylation decrease in both homoeologs of *TaDOS* in ETW. The scales are 0–1 for CG and CHG and 0–0.05 for CHH. **d** Expression levels (FPKM) of *TaDOS* homoelogs

In ETW, CG, CHG, and CHH hypo-DMRs overlap with 1520, 9612, and 964 genes in their promoter regions, respectively (Additional file 2: Table S6). These genes are overrepresented in GO terms of metabolism and translation-related processes, including photosynthesis, metabolic process, and translation (Fig. 3a, hypergeometric test, *P* < 0.05). The function of photosynthesis in seed development is unclear. In *Arabidopsis*, photosynthetic genes are expressed in the peripheral endosperm [20], which may suggest a role for photosynthetic activities in the endosperm and seed coat development. Notably, most hyper- or hypo-DMRs were associated with only one homoeolog (Fig. 3b), implying a role for DNA methylation in biased expression of homoeologs. The fraction of DMRs associated with both A and B homoeologs was relatively small, albeit a slightly higher fraction (696, ~ 23%) of CHG hypo-DMRs with both homoeologs. *TaDOS* is an example. *OsDOS*, a rice homolog, is known to regulate spike development and fertility [21]. Both A and B homoeologs of *TaDOS* were demethylated and expressed at higher levels in the ETW than in RHW and NHW (Fig. 3c, d), suggesting a potential role for *TaDOS* in seed phenotypic changes in the extracted tetraploid wheat.

### Unique genomic regions of demethylation and TE activation in ETW

**Fig. 4:**
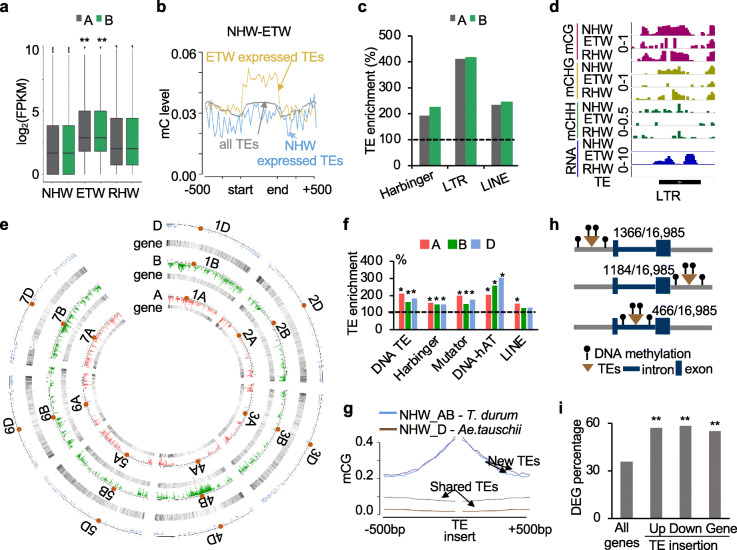
DNA methylation variation, TE expression, and expansion during wheat polyploidization. **a** Expression levels of differentially expressed TEs in NHW, ETW, and RHW (Wilcoxon rank-sum test with double asterisks indicating a significant level of *P* < 0.01). **b** Methylation changes of TEs that were expressed specifically in the ETW (yellow line) and NHW (blue line) using all TEs (gray line) as a control. **c** Overrepresentation of expressed TEs in three TE groups (hypergeometric test, *P* < 0.05). **d** An example of TE with increased expression and reduced DNA methylation levels. The box indicates the DMR region. The scales are 0–1 for CG and CHG, 0–0.05 for CHH, and 0–50 for FPKM. **e** Distribution of TEs in NHW relative to NTW and D-genome diploid in A (red), B (green), and D (blue) subgenomes. **f** Overrepresentation of five TE groups in A (red), B (green), and D (blue) subgenomes. **g** CG methylation density plots showing an increase in the upstream and downstream regions of the TEs in NHW relative to NTW (blue) and D-genome diploid (purple). Shared TEs in NHW and NTW are used as a control. **h** Distribution of TEs in upstream, downstream, and gene body. **i** Percentage of DEGs with TE insertions in upstream, downstream, and gene body using all expressed genes as a control. Double asterisks indicate a significance level of *P* < 0.01 (hypergeometric test)

Genome-wide hypomethylation could activate TEs in the ETW. Indeed, among 5117 differentially expressed TEs between ETW and RHW (Fig. 4a), 36.3% (1860) and 12.3% (628) TEs were expressed specifically in ETW and NHW, respectively (Fig. 4b). The TEs that were activated in ETW were dramatically demethylated, whereas the methylation levels were similarly low in the coding regions among all TEs and NHW-expressed TEs (Fig. 4b). Among ETW-activated TEs, DNA-Harbinger, LTR, and LINE TEs were overrepresented (Fig. 4c, hypergeometric test, *P* < 0.05). For example, a LTR TE in the ETW was hypomethylated in CG, CHG, and CHH sites and highly expressed (Fig. 4d). These data suggest that TEs are demethylated and activated in ETW, which may be methylated and silenced in the natural hexaploid wheat.

Next, we examined if ETW and NTW (*T. durum*) share similar methylation profiles since they have similar genomic composition (AABB) and conserved karyotypes (Additional file 1: Figure S2) [13]. There were 229,739 CG, 567,555 CHG, and 64,184 CHH DMRs between *T. durum* and NHW (Additional file 1: Figure S4a), and the number was not significantly different between hyper- and hypo-DMRs. Although these DMRs were evenly distributed between A and B subgenomes (Additional file 1: Figure S4a), only 2~12% of DMRs overlapped between ETW and *T. durum* (Additional file 1: Figure S4b). Moreover, different TEs were expressed specifically in ETW or *T. durum*. These data may suggest that genome-wide demethylation and TE activation in the ETW occur at the genomic regions that are different from those in the natural tetraploid wheat.

Using the resequencing data, we identified subgenomic TEs (see the “Methods” section), including 4933 (A subgenome), 8005 (B subgenome), and 4047 (D subgenome), which were present only in the natural hexaploid wheat but absent in the natural tetraploid wheat or D-genome diploid (Fig. 4e and Additional file 2: Table S7). These TEs were distributed predominately in chromosome arms and sparsely in pericentromeric regions (Fig. 4e), which were different from distribution patterns of other shared TEs (present in all NHW, NTW, and D-genome diploid lines) (Additional file 1: Figure S3b). Many of these TEs were associated with genes, suggesting that hexaploid bread wheat may have shaped DNA methylation and gene expression diversity for selection, adaptation, and domestication. Two exceptions exist for chromosomes 4B and 7B, where new TEs are present mostly in centromeric regions (Fig. 4e).

These TEs are enriched for DNA transposons, including Harbinger, Mutator, and hAT (Fig. 4f, hypergeometric test, *P* < 0.05). In addition, DNA methylation levels of neighboring regions in these TEs were substantially increased compared to the shared TEs (Fig. 4g, Wilcoxon rank-sum test, *P* < 0.05). Among them, 1366 (A), 1184 (B), and 466 (D) TEs were present in the upstream (5′), downstream (3′), and the gene-body regions, respectively (Fig. 4h). The fraction of DEGs between the hexaploid wheat and its diploid and tetraploid relatives was significantly higher in the TE regions than that in all genes (Fig. 4i, hypergeometric test, *P* < 0.05) (DEGs/all expressed genes). These TE-associated genes were overrepresented in cellular protein modification process, nucleotide binding, and carbohydrate binding (Additional file 1: Figure S4c, hypergeometric test, *P* < 0.05). For example, TraesCS2B01G454000 has a TE in its promoter and is hypermethylated and expressed at low levels (Additional file 1: Figure S4d). These TEs present in genic regions may induce DNA methylation through RNA-directed DNA methylation [22], which in turn suppresses expression of TE-associated genes in the hexaploid wheat.

### Decreased H3K9me2 and increased histone expression levels in ETW

**Fig. 5:**
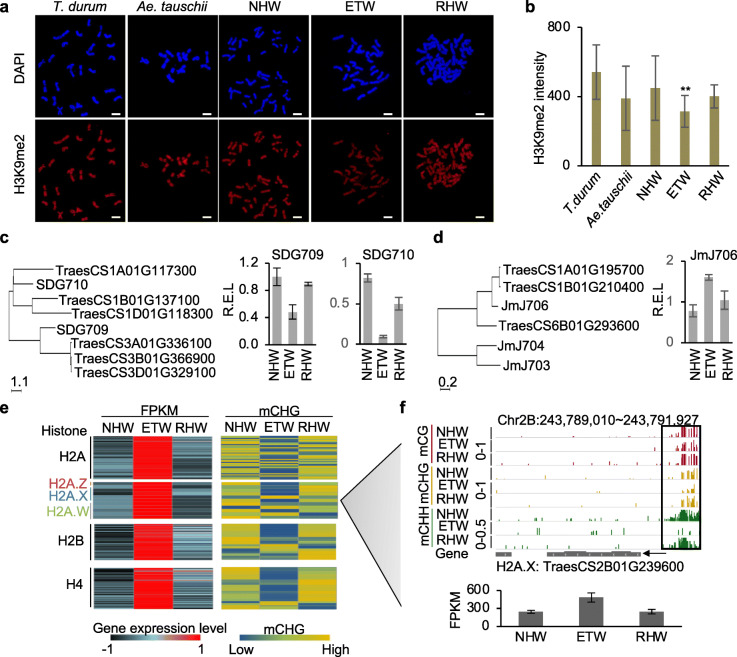
Upregulation of H3K9me2 demethylase genes and histone genes and downregulation of H3K9me2 methyltransferase genes in ETW. **a** Representative immunofluorescence images with antibodies against H3K9me2 in metaphase cells of five wheat species. Scale bar = 10 μm. **b** Relative immunofluorescent intensities of H3K9me2 per chromosome for samples in **a**. Error bars indicate standard deviation with double asterisks showing a significance level of *P* < 0.01. **c** Two H3K9me2 histone methyltransferase genes *SDG710* and *SDG709* in rice and homoeologs in wheat with their expression levels in NHW, ETW, and RHW. **d** Histone H3K9me2 demethylase genes (JMJ706 and its related genes) in rice and wheat with expression levels in NEW, ETW, and RHW. **e** Heatmaps show gene expression levels (left) and CHG methylation levels in promoter regions (right) of upregulated histone H2A, H2B, and H4 genes in ETW. **f** An example showing hypomethylation and upregulation of an H2A gene in ETW. The box indicates the DMR region. Scales are 0–1 for CG and CHG and 0–0.5 for CHH

Genome-wide CHG demethylation in ETW may suggest a role for H3K9me2, as it is highly correlated with CHG methylation in plants [23]. Indeed, we found that overall H3K9me2 levels were reduced in ETW and reverted to the normal level in RHW (Fig. 5a, b, *t* test, *P* < 0.05). The reduced levels of H3K9me2 in ETW were associated with the upregulation of H3K9me2 demethylase genes such as *SDG709* and *SDG710* and downregulation of H3K9me2 methyltransferase genes such as *JMJ706* (Fig. 5c, d and Additional file 1: Figure S5a). Moreover, many genes encoding histone H2A, H2B, and H4 were also upregulated in ETW (Fig. 5e), which correlated with decreased CHG methylation levels in their putative promoter regions. In plants, there are several histone H2 variants, including H2A.Z, H2A.X, and H2A.W [24]. DNA methylation can influence chromatin structure and affect gene silencing by excluding histone H2A.Z and H2A.Z [25]. We found that the expression of several histone H2 variants was also upregulated and correlated with DNA methylation changes in their putative promoter regions (Fig. 5e and Additional file 1: Figure S5b). For example, *TraesCS2B01G239600*, encoding H2A.X, was demethylated at its putative promoter region and expressed at higher levels in ETW than in NHW and RHW (Fig. 5f). These data suggest that overall chromatin modifications including decreased H3K9me2 levels and increased histone variant distribution may contribute to demethylation in ETW after genome separation from natural hexaploid wheat.

## Discussion

Hexaploid wheat was formed 8000–10,000 years ago by hybridizing a tetraploid wheat with a D-genome diploid coupled with genome doubling [8]. After polyploidization, three sets of subgenomes (A, B, and D) could have experienced genetic and epigenetic modifications. In newly formed allotetraploid wheat, sequence elimination and DNA methylation can occur rapidly [26], which lead to expression bias among homoeologs [27]. Estimates indicate that ~ 30% homoeologous triads in hexaploid bread wheat display biased expression [28], and some genes with the expression bias are associated with DNA methylation and TE variation within promoter regions of those homoeologs [29]. Notably, homoeologous chromosomes in the ETW (AABB) pair normally during meiosis and show little genetic variation (Additional file 1: Figure S2) [13]. This may suggest more epigenetic changes than genetic changes in the ETW after genome separation, as previously reported [15]. It is likely that epigenetic modifications have been reprogrammed after the formation of hexaploid wheat and during 10,000 years of evolution and domestication. One such modification is DNA methylation, which has been reduced in ETW, compared to NHW and NTW. This global hypomethylation is dependent on the *trans*-effect by the D subgenome. When a D-genome diploid is merged with the ETW to form a hexaploid wheat, the methylation levels return to the normal level. This remethylation at the genome-wide level is unlikely from spontaneous mutants during separation and merger of the D genome in hexaploid wheat. Interspecific hybridization may induce DNA methylation changes that are stably maintained in the hexaploid wheat, as observed in five allotetraploid cotton species [4, 30, 31]. Further studies using additional sets of extracted and resynthesized wheat and/or other polyploid plants should address if this demethylation-remethylation is a rule or exception for the genome separation and merger during polyploidization.

Genome-wide demethylation occurs in the endosperm, as observed in *Arabidopsis* [32, 33] and rice [34, 35]. In wheat, this demethylation in the endosperm is more profound in the ETW than in the NTW and can be reversed after the addition of the D genome, suggesting a potential effect of the D genome on remethylation in the hexaploid wheat. However, we cannot rule out other possibilities such as imprinting and activation of other factors in the resynthesized hexaploid wheat, which may also mediate methylation changes in the endosperm. In addition, it will be interesting to test if this type of methylation changes also occurs in other tissues and organs.

Notably, genome-wide DNA methylation reprogramming that occurred in the ETW (AABB separated from the hexaploid, AABBDD) is different from the overall methylation distribution in natural tetraploid wheat (AABB). Only 2~12% DMRs (relative to NHW) overlap between ETW and *T. durum*. This suggests that interactions between AB and D subgenomes during interspecific hybridization and polyploidization could induce epigenetic changes in genomic regions that are different from those present in the progenitor-like AABB genomes. Alternatively, the extant tetraploid wheat (*T. durum*) may not be the tetraploid donor of hexaploid wheat, because the exact progenitor-like tetraploid and diploid species are unknown [8] or became extinct. Interestingly, the DNA methylation changes in the ETW after separation from the D subgenome could be restored by the addition of the D genome. This may indicate inter-dependency of epigenetic modifications between three subgenomes in the allohexaploid wheat. The reduced DNA methylation in the ETW (AABB) after separation of DD is similar to the phenomenon of *trans*-acting demethylation (TAdM), as observed in *Arabidopsis* [36]. These *trans*-acting factors could be small RNAs [7].

TEs can represent ~ 40% of genome in humans and over 80% in plants including wheat [11]. Although TEs can determine genome size, generate mutations and chromosome rearrangements, and regulate gene expression, the majority of them are methylated and remain in heterochromatic regions [37]. Global demethylation, especially in CHG hypomethylation, contributes to the activation of TEs in ETW, which can affect genome stability, gene expression, and phenotypic changes. Loss of CHG methylation in the rice *oscmt3a* mutant induces activation of TEs including Copia and Gypsy elements [38]. In *Ae. tauschii*, TEs are associated with about half of the genes, and such genes are hypermethylated and expressed at lower levels than those without TEs [39]. Although the genic regions are conserved among three subgenomes in hexaploid wheat [11], there is a massive turnover of TEs since A, B, and D diverged [40]. Using the resequencing data, we found TE distributions more in the B subgenome than A and D subgenomes, consistent with the subgenome size, where the B subgenome is nearly twice the size of A and D subgenomes [11]. Interestingly, these TEs may regulate DNA methylation and expression of TE-associated genes during the evolution and domestication of hexaploid bread wheat.

In plants, H3K9me2 marks are associated with CHG methylation through CMT3-mediated pathway [23], and mutation in *SDG714* encoding a H3K9 methyltransferase leads to decreased H3K9 methylation as well as CG and CHG methylation in rice [41]. Consistent with this finding, in ETW where CHG methylation is reduced, H3K9me2 demethylase genes are upregulated, while H3K9me2 methyltransferase genes are downregulated in ETW. As a result, overall H3K9me2 levels on homoeologous chromosomes are generally decreased in ETW. Although the cause and effect remain to be tested, these results indicate concerted epigenetic changes between H3K9me2 and DNA demethylation in ETW. Consistent with this notion, other chromatin factors such as core histone H2A, H2B and H4, and histone H2A variant genes are hypomethylated in their promoter regions and upregulated in the ETW. Together, these data suggest that intergenomic interactions during genome merger and separation may induce chromatin and DNA methylation changes that are stably maintained in allopolyploids. These concerted epigenetic changes could shape gene expression diversity, TE expansion, and genome stability, which may contribute to the evolution and domestication of polyploid wheat and other crops.

## Conclusions

Genome separation and merger in hexaploid bread wheat can provoke dynamic and reversible changes in DNA methylation and histone modifications, suggesting pervasive epigenetic changes induced in allopolyploids such as wheat and cotton. These changes accompany altered TE activity and gene expression, which may lead to phenotypic variation for selection, adaptation, and domestication. Our results provide unique insights into epigenetic roles in genome merger, separation, and evolution of polyploid wheat and other crops.

## Methods

### Plant materials and DNA/RNA extraction

Plant materials included extracted tetraploid wheat (ETW, AABB), natural hexaploid bread wheat (NHW, *T. aestivum* L. cv Canthach, AABBDD), natural tetraploid wheat (NTW, *T. turgidum* L. subsp. *durum*, AABB), *Ae. tauschii* subsp. *strangulata* (line RL5288, DD), and resynthesized hexaploid wheat (RHW, AABBDD). Starch content was analyzed with three biological replications from 20 seeds at 6, 9, 12, and 15 days after pollination (DAP) and maturing stage using the protocol described in the Total Starch Assay Kit (K-TSTA) (Megazyme, Co. Wicklow, Ireland). Endosperm at 6 DAP was manually dissected and used for DNA and RNA extraction [42]. Trefoil stage leaves of *T. durum*, *Ae. tauschii*, ETW, and NHW were used for DNA extraction and resequencing. Genomic resequencing, whole-genome bisulfite sequencing, and RNA-seq with three biological replications were performed following the published methods [4].

### Analysis of karyotypes, DNA methylation, and H3K9me2 immunofluorescence

The protocol for fluorescence in situ hybridization (FISH) was adopted as previously described [43]. For FISH, repetitive DNA sequences *pSc119.2* and a microsatellite (GAA)n oligonucleotide were labeled with Chroma Tide Alexa Fluor 488-5-dUTP (C11397, Invitrogen, Eugene, USA), and *pAs1* was labeled with Texas Red-5-dCTP (NEL 426, Perkin-Elmer, Boston, USA). Anti-methylcytosine antibodies (1982501, Millipore, Temecula, USA) and anti-dimethyl-Histone H3 (Lys9) antibodies (07-441, Millipore, Temecula, USA) were used for immunofluorescence analysis using published protocols [5, 42]. Slides were examined with an Olympus BX61 fluorescence microscope and digitally photographed. Immunofluorescence values of DNA methylation and H3K9me2 were estimated from homoeologous chromosomes of 30 cells using an Olympus cellSens Dimension and were averaged by the number of chromosomes in a given genotype.

### Resequencing and identification of SNPs, Indels, CNVs, and TEs

Sequencing reads (150-bp paired-end) of *T. durum*, *Ae. tauschii*, ETW, and NHW were trimmed using NGSQCToolkit_v2.3 to remove low-quality reads [44]. High-quality reads were mapped onto the genome of *T. aestivum* (Chinese Spring, WGAv1) [11] using BWA (BWA-MEM algorithm) with default parameters [45]. Read pairs with a mapping quality lower than 10 were removed. Single nucleotide polymorphisms (SNPs) and insertion-deletions (Indels, 2–50 bp) were identified using samtools, with quality scores > 100, depth > 10, and 90% or more read coverage. SNPs were then substituted in the corresponding reference genomes of each genotype for further analysis. ETW combined with the *Ae. tauschii* sequence for the analysis of RHW. Copy number variants (CNVs) (deletions and duplications) were analyzed using delly [46] and lumpy [47], each with 5 or more supporting paired-end reads and MAPQ > 20. CNVs detected by delly and lumpy methods were combined into one file using the loci with 80% reciprocal overlaps.

The TEs present in hexaploid wheat but not in *Ae. tauschii* and *T. durum* were identified using the published methods [48]. We mapped the reads of *Ae. tauschii* and *T. durum* onto D and AB subgenomes of *T. aestivum* (Chinese Spring, WGAv1), respectively. Split and discordant reads were extracted to identify absence variants of TEs. A split read was defined to contain the separation point, with the segment separated by more than 2-kb distance. Discordant reads were defined as mate-paired reads that were aligned to the reference genome with more than 2-kb distance. We first identified annotated TEs in the reference genome that were spanned by split and discordant reads. Among these TEs, we then identified the candidates of TE absence variants using the criteria of a split or discordant read spanning at least 80% of the TE sequence, and the sequencing depth within the spanned region is < 10% of the 2-kb flanking sequence. In addition, we extracted NHW resequencing reads that were not aligned to the genomes of *Ae. tauschii* and *T. durum* and mapped these reads to the genome of *T. aestivum*. Finally, the candidates of TE absence variants with 80% or more regions covered by resequencing reads of NHW were identified as absence variants of TEs in *Ae. tauschii* and *T. durum* relative to hexaploid wheat.

### RNA-seq and gene expression analysis

After removing low-quality reads, RNA-seq reads were aligned with default parameters using TopHat [49] to the corresponding references of each genotype, retaining the uniquely mapped reads with both pairs mapped onto the same chromosome. For interlibrary comparison, RNA-seq reads were normalized to fragment per kiobase per million (FPKM) using Cufflinks [49], and the genes with FPKM > 1 in at least two biological replications were used for further analysis. The reads from two compared groups were normalized by their respective size factors, which were analyzed by DESeq package (differential expression analysis for sequence count data) (parameter: estimateSizeFactors). Fold-cut and FDR values were 2 and FDR < 0.05 for differentially expressed genes (DEGs) and 1.5-fold and FDR < 0.05 for differentially expressed TEs.

Gene Ontology (GO) classification was performed using BLAST software, and GO enrichment analysis was implemented by Blast2go [50]. GO term analysis was performed using GOSlim using all genes in each subgenome or all subgenomes as a control. Hypergeometric test was used for statistical significance tests (*P* < 0.05).

### Identification of homoeologs among A, B, and D subgenomes

Homoeologous genes between each pair of A, B, and D subgenomes in Chinese Spring [11] were identified as previously described [42]. Briefly, coding sequences (CDSs) of the two subgenomes were aligned reciprocally with a BLASTN cutoff value of *e*^−10^ [51]. Gene pairs with over 90% sequence identity and an overall length of orthologous regions larger than 60% of the CDSs in both subgenomes were defined as homoeologous genes. The one-to-one reciprocal homoeologs were regarded as dyad. Dyads present in all three comparisons were considered as triad.

### Whole-genome DNA methylation analysis

Bisulfite sequencing reads of NHW, ETW, and RHW were mapped to SNP-corrected Chinese Spring genome sequence (WGAv1). Reads of *Ae. tauschii* and *T. durum* were mapped to corresponding SNP-corrected genome sequences, respectively*.* Read mapping was performed using Bismark (parameter: bowtie2 –N 0) [52]. SNPs in the regions with CNVs and Indels were excluded from the analysis. Only the cytosines covered by at least three reads in all compared wheat materials were used for further analysis. Cytosines with *P* value below 10^−5^ were identified as methylcytosines (binomial distribution). Genome-wide DNA methylation was calculated using the proportion of methylcytosines in all reads covered cytosines in every chromosome window (1 Mb) and shown using circos plots (http://circos.ca/).

Differentially methylated regions (DMRs) between two genotypes were identified using a published workflow by 200-bp sliding-window [4]. The methylation level was calculated by (mC)/(mC + non-mC), in which mC and non-mC refer to the number of methylated and unmethylated cytosines in one region. The mean methylation level was estimated for each window. A window with both two materials containing 16, 16, and 64 or more reads in CG, CHG, and CHH content was kept for further analysis. Statistical significance for DMRs in each window was weighted using Fisher’s exact test with corrected *P*_adj_ < 0.05. Finally, all significant windows were filtered by the following criteria: the methylation level difference between two alleles was higher than 0.5 (50%), 0.3 (30%), and 0.1 (10%) in CG, CHG, and CHH contexts, respectively.

We used a sliding-window approach, dividing the region into 60 windows to show DNA methylation level changes, to display distribution of DNA methylation levels in the gene body with 2000-bp up- and downstream regions and TEs with 500-bp up- and downstream regions. Integrative Genomics Viewer (IGV) [53] was used to show the distribution of DNA methylation and gene expression levels for specific examples.

### Quantitative RT-PCR analysis

After DNase treatment, total RNA (1 μg) was reverse-transcribed to cDNA using HiScript II Q RT SuperMix for qPCR (Vazyme R223-01). The cDNA was used as the template for qRT-PCR using AceQ qPCR SYBR Green Master Mix (Q111-02, Vazyme, Nanjing, China). A housekeeping gene encoding glyceraldehyde3-phosphate dehydrogenase (*GADPH*) was used as a control for expression analysis in *Triticeae* [14]. Primer pairs for qRT-PCR analysis are listed in (Additional file 2: Table S8).

## Supplementary Information

**Additional file 1: Figure S1.** Extracted tetraploid wheat (ETW) shows decreased starch content, smaller starch granule, and reduced fertility. (a) Images showing pollen fertility by iodine staining (top panel) and starch structure by scanning electron microscopy (bottom panel). S refers to starch granules. Materials are *T. durum* (AABB), *Ae. Tauschii* (DD), natural hexaploid wheat (NHW, AABBDD), ETW (AABB), and resynthesized hexaploid wheat (RHW, AABBDD). Scale bars = 100 μm (top images) and 15 μm (bottom images). (b) Developing seed morphology at 6 days after pollination (DAP) in the same set of lines as in (a). Scale bar = 0.5 cm for all images. (c, d) Total starch content (mg/100 mg) (c) and thousand-kernel weight (grams) (d) of the same set of lines as in (a). Error bars indicate standard deviation of three biological replicates with three asterisk showing a statistical significance level of *P* < 0.0001. **Figure S2.** Karyotypes of five wheat species. Karyotypes of *Ae. tauschii* (DD), *T. durum* (AABB), ETW, NHW and RHW. The probes used in FISH are *pSc119.2* (green), *pAs1* (red) and (GAA) n (yellow). Scale bar = 10 μm. **Figure S3.** The DNA methylation levels between ETW (AABB) and NHW (AABBDD). (a) Fraction of methylated cytosine (mC) in *T. durum* (blue), *Ae. tauschii* (purple), NHW (green), ETW (yellow) and RHW (pink). One asterisk indicates a statistical significance level of *P* < 0.05. (**b**) Circos plot showing CG, CHG and CHH methylation levels in the B subgenome of NHW, ETW and RHW. Blue and gray circos indicate gene and TE densities, respectively. Scales are 0–1 for CG and CHG, 0–0.05 for CHH. **Figure S4.** DNA methylation variation and TE expansion during wheat polyploidization. (a) DMRs of A, B or D subgenome between *T. durum*, *Ae. tauschii* (DD) and NHW (AABBDD). Scales are 0–1 for CG and CHG, 0–0.05 for CHH. (b) Comparative analysis of DMRs between ETW and NHW with those between *T. durum* and NHW. (c) Gene ontology (GO) representation of the genes with the TEs inserted in the A (red), B (green) or D (blue) subgenome. CPMP: cellular protein modification process; NB: nucleotide binding; KA: kinase activity; TA: transferase activity; CB: carbohydrate binding. Hypergeometric test (*P* < 0.05) was used for statistical significance analysis. (d) An example of the TE-associated gene that shows expression changes. The box region indicates the region of TE insertion and DNA methylation changes. Scales are 0–1 for CG and CHG, 0–0.5 for CHH. (e) The TE was present in NHW (top) and related to expression changes (fragments per kilobase pairs per million, RPKM) in the TE-associated gene (bottom). **Figure S5.** Expression and phylogenetic analyses of DNA and histone methylation-related genes and H2A families in ETW and NHW. (a) Expression levels (logRPKM) of putative DNA methylation-related genes (*MET1b*, *CMT3b*, *DDM1a*, *CMT2*, *ROS1*, and *DNG703*) and H3K9me2 writers (*SDG701*, *SDG703*, *SDG704*, *SDG710*, *SDG723*, and *SDG726*) and erasers (*JmJ706*) (b) Phylogenetic relationships of H2A gene families in hexaploidy wheat.

**Additional file 2.** Supplementary Tables.

## Data Availability

Sequencing data are deposited under accession number CRA002143 in Genome Sequence Archive in BIG Data Center https://bigd.big.ac.cn/gsa [54]. The use of plant materials follows local, national, or international guidelines and legislation and the required or appropriate permissions and/or licenses for the study.
