# Supplementary material for: Dynamic and reversible DNA methylation changes induced by genome separation and merger of polyploid wheat
Source: BMC Biol. 2020 Nov 20;18:171. doi: 10.1186/s12915-020-00909-x (PMC7679994; doi:10.1186/s12915-020-00909-x)
Supplement: Supplementary file 1 — Additional file 1: Figure S1. Extracted tetraploid wheat (ETW) shows decreased starch content, smaller starch granule, and reduced fertility. (a) Images showing pollen fertility by iodine staining (top panel) and starch structure by scanning electron microscopy (bottom panel). S refers to starch granules. Materials are T. durum (AABB), Ae. Tauschii (DD), natural hexaploid wheat (NHW, AABBDD), ETW (AABB), and resynthesized hexaploid wheat (RHW, AABBDD). Scale bars = 100 μm (top images) and 15 μm (bottom images). (b) Developing seed morphology at 6 days after pollination (DAP) in the same set of lines as in (a). Scale bar = 0.5 cm for all images. (c, d) Total starch content (mg/100 mg) (c) and thousand-kernel weight (grams) (d) of the same set of lines as in (a). Error bars indicate standard deviation of three biological replicates with three asterisk showing a statistical significance level of P < 0.0001. Figure S2. Karyotypes of five wheat species. Karyotypes of Ae. tauschii (DD), T. durum (AABB), ETW, NHW and RHW. The probes used in FISH are pSc119.2 (green), pAs1 (red) and (GAA) n (yellow). Scale bar = 10 μm. Figure S3. The DNA methylation levels between ETW (AABB) and NHW (AABBDD). (a) Fraction of methylated cytosine (mC) in T. durum (blue), Ae. tauschii (purple), NHW (green), ETW (yellow) and RHW (pink). One asterisk indicates a statistical significance level of P < 0.05. (b) Circos plot showing CG, CHG and CHH methylation levels in the B subgenome of NHW, ETW and RHW. Blue and gray circos indicate gene and TE densities, respectively. Scales are 0–1 for CG and CHG, 0–0.05 for CHH. Figure S4. DNA methylation variation and TE expansion during wheat polyploidization. (a) DMRs of A, B or D subgenome between T. durum, Ae. tauschii (DD) and NHW (AABBDD). Scales are 0–1 for CG and CHG, 0–0.05 for CHH. (b) Comparative analysis of DMRs between ETW and NHW with those between T. durum and NHW. (c) Gene ontology (GO) representation of the genes with the TEs inserted in the [file 12915_2020_909_MOESM1_ESM.pdf]

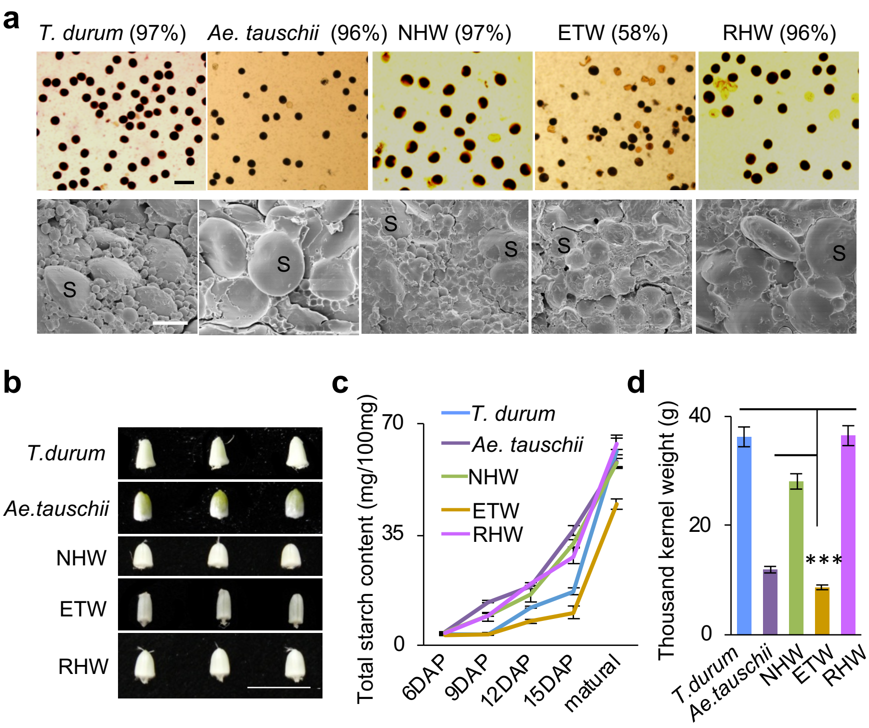

Figure S1. Extracted tetraploid wheat (ETW) shows decreased starch content, smaller starch granule, and reduced fertility. (**a**) Images showing pollen fertility by iodine staining (top panel) and starch structure by scanning electron microscopy (bottom panel). S refers to starch granules. Materials are *T. durum* (AABB), *Ae. Tauschii* (DD), natural hexaploid wheat (NHW, AABBDD), ETW (AABB), and resynthesized hexaploid wheat (RHW, AABBDD). Scale bars=100 μm (top images) and 15 μm (bottom images). (**b**) Developing seed morphology at 6 days after pollination (DAP) in the same set of lines as in (**a**). Scale bar=0.5 cm for all images. (**c, d**) Total starch content (mg/100mg) (**c**) and thousand-kernel weight (grams) (**d**) of the same set of lines as in (**a**). Error bars indicate standard deviation of three biological replicates with three asterisk showing a statistical significance level of $P<0.0001$.

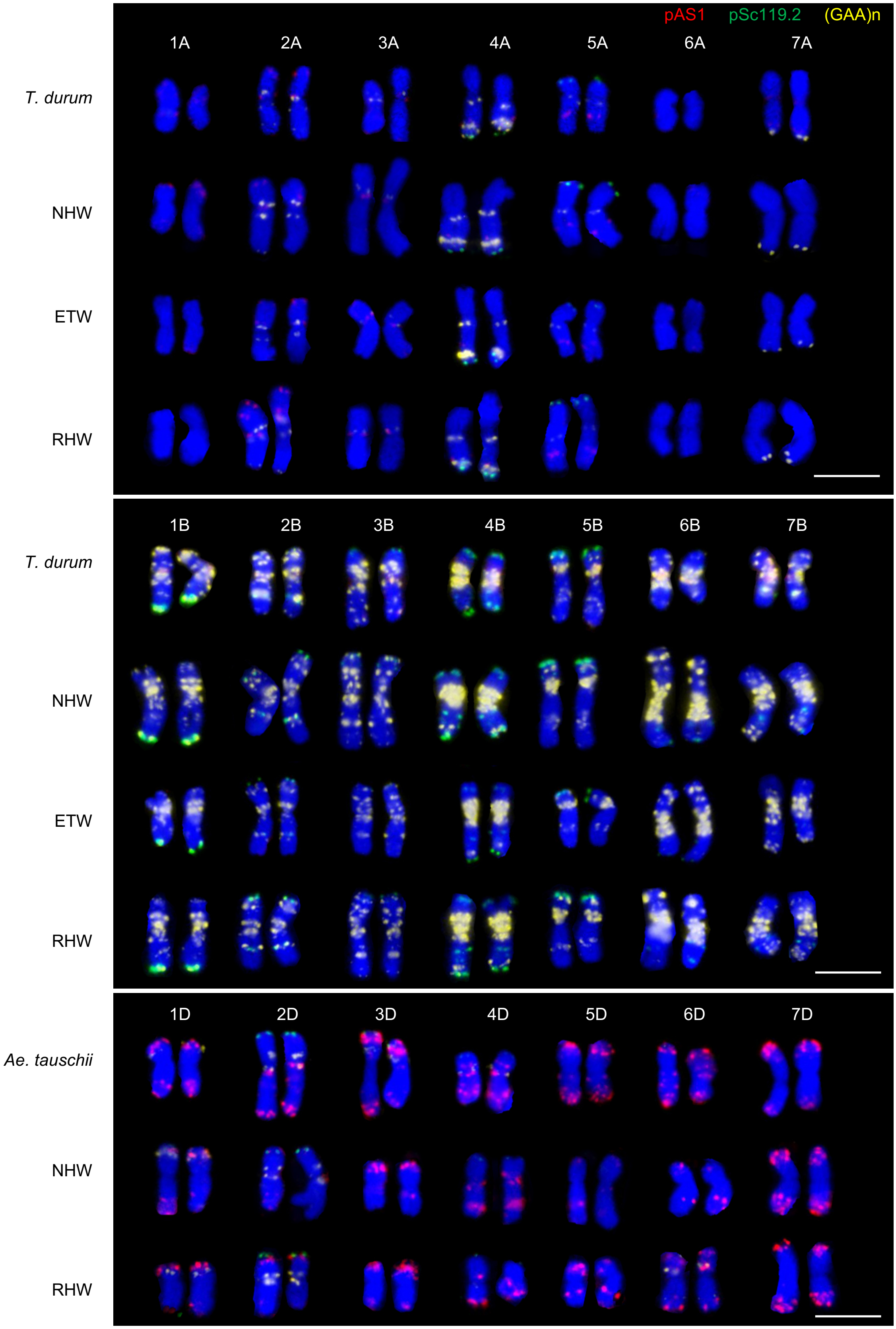

Figure S2. Karyotypes of five wheat species. Karyotype analysis of *Ae. tauschii* (DD), *T. durum* (AABB), ETW (AABB), NHW (AABBDD) and RHW (AABBDD). The probes used in FISH are *pSc119.2* (green), *pAs1* (red) and (GAA)n (yellow). Scale bars=10 μm for all images.

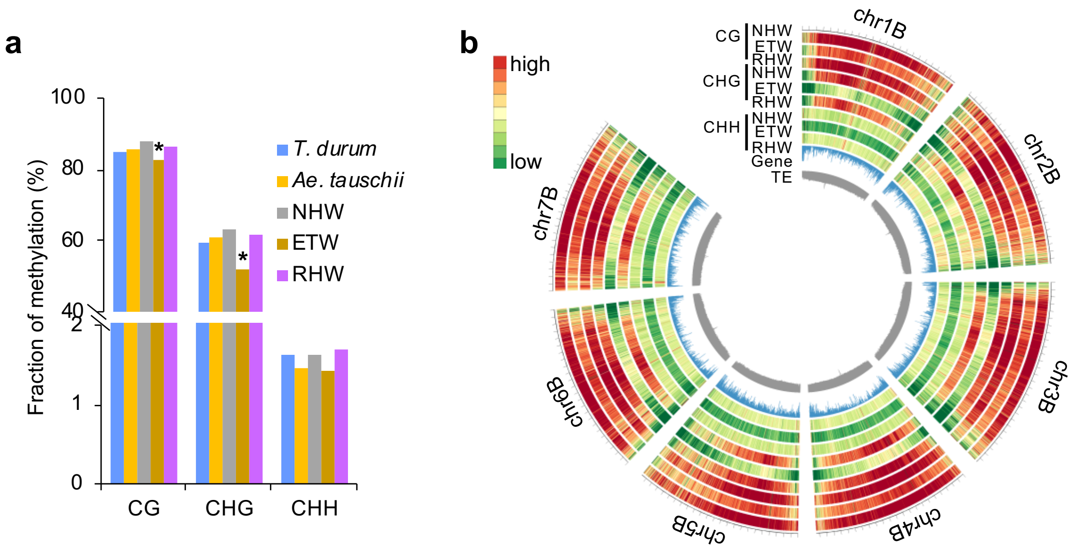

Figure S3. The DNA methylation levels between ETW (AABB) and NHW (AABBDD). (**a**) Fraction of methylated cytosine (mC) in *T. durum* (blue), *Ae. tauschii* (purple), NHW (green), ETW (yellow) and RHW (pink). One asterisk indicates a statistical significance level of $P<0.05$. (**b**) Circos plot showing CG, CHG and CHH methylation levels in the B subgenome of NHW, ETW and RHW. Blue and grey circos indicate gene and TE densities, respectively. Scales are 0-1 for CG and CHG, 0-0.05 for CHH.

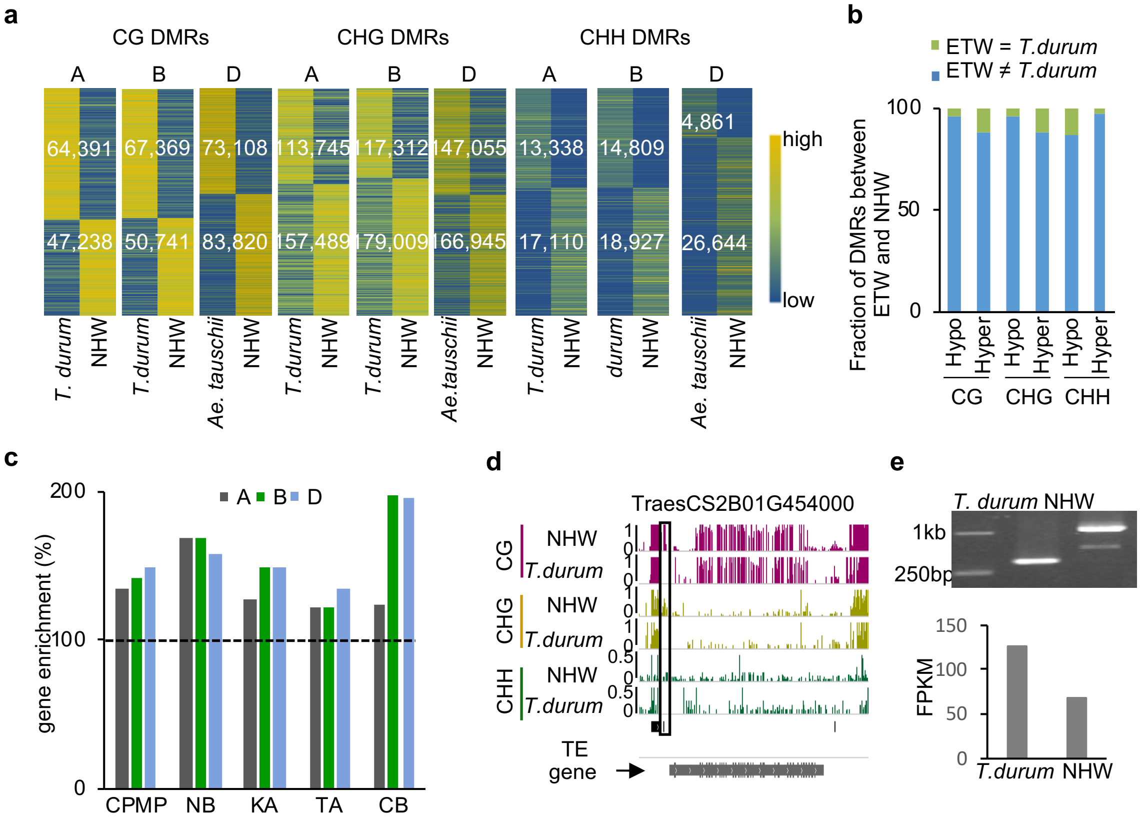

Figure S4. DNA methylation variation and TE expansion during wheat polyploidization. (**a**) The DMRs between *T. durum*, *Ae. tauschii* and NHW in A, B and D subgenomes. The scales are 0-1 for CG and CHG, 0-0.05 for CHH. (**b**) DMRs between ETW and NHW overlapped with those between *T. durum* and NHW. (**c**) GO representation of genes with TEs inserted in A (red), B (green) and D (blue) subgenomes. CPMP: cellular protein modification process; NB: nucleotide binding; KA: kinase activity; TA: transferase activity; CB: carbohydrate binding. (Hypergeometric test, $P < 0.05$). (**d**) An example of gene expression changed with TE insertion. The box indicates TE insertion locus in NHW and DNA methylation level changed region. (**e**) The TE was present in NHW (top) and related to expression changes (fragments per kilobase pairs per million, RPKM) in the TE-associated gene (bottom).

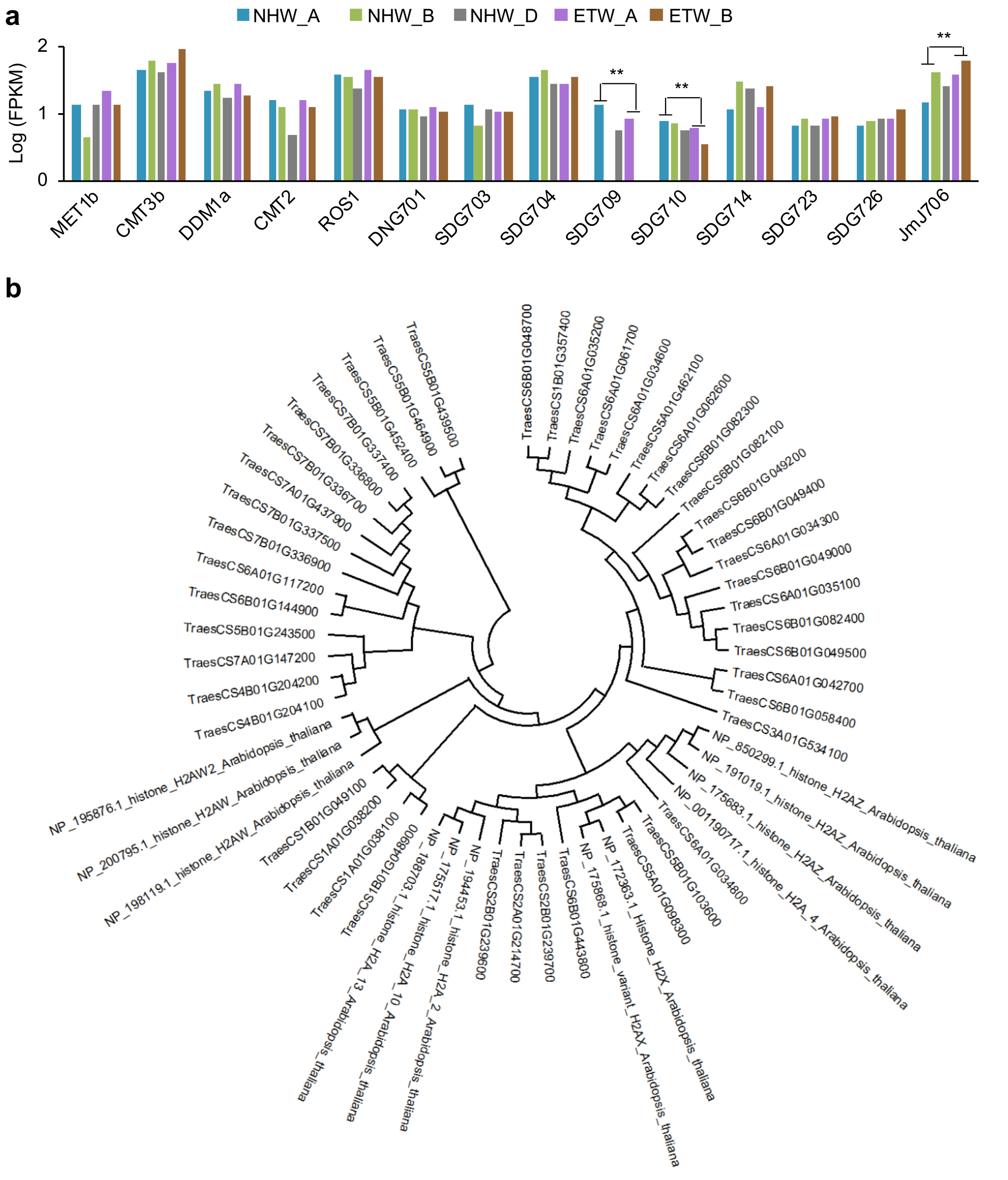

Figure S5. Expression and phylogenetic analyses of DNA and histone methylation-related genes and H2A families in ETW and NHW. (a) Expression levels (LogRPKM) of putative DNA methylation-related genes (*MET1b*, *CMT3b*, *DDM1a*, *CMT2*, *ROS1*, and *DNG703*) and H3K9me2 writers (*SDG701*, *SDG703*, *SDG704*, *SDG710*, *SDG723*, and *SDG726*) and erasers (*JmJ706*) (**b**) Phylogenetic relationships of H2A gene families in hexaploidy wheat.
